# Real-World and Clinical Implications of Patient Education, Lifestyle and Treatment Adherence in Romanian Diabetes Care: An Observational Study

**DOI:** 10.3390/jcm14207171

**Published:** 2025-10-11

**Authors:** Ozana-Andreea Măriuț, Ana Flavia Burlec, Irina Macovei, Cornelia Mircea, Mădălina Elena Datcu, Monica Hăncianu, Andreia Corciovă

**Affiliations:** 1 Department of Drug Analysis, Faculty of Pharmacy, Grigore T. Popa University of Medicine and Pharmacy, 16 University Street, 700115 Iasi, Romania; stoleruozana@gmail.com (O.-A.M.); irina-macovei@umfiasi.ro (I.M.); maria.corciova@umfiasi.ro (A.C.); 2 Department of Pharmaceutical Biochemistry and Clinical Laboratory, Faculty of Pharmacy, Grigore T. Popa University of Medicine and Pharmacy, 16 University Street, 700115 Iasi, Romania; cornelia.mircea@umfiasi.ro; 3 Department of Biomedical Sciences, Grigore T. Popa University of Medicine and Pharmacy, 700115 Iasi, Romania; madalina.dtc@gmail.com; 4 Department of Pharmacognosy, Faculty of Pharmacy, Grigore T. Popa University of Medicine and Pharmacy, 16 University Street, 700115 Iasi, Romania; monica.hancianu@umfiasi.ro

**Keywords:** diabetes mellitus, treatment adherence, lifestyle, self-management, complications, digital health, community pharmacies, observational study

## Abstract

**Background/Objectives:** Diabetes mellitus is a major global health concern requiring both preventive strategies and patient-centered clinical management. This study evaluated knowledge, lifestyle behaviors, treatment adherence and the use of digital tools among Romanian patients with diabetes in a real-world setting. **Methods:** A cross-sectional observational study was conducted on 100 patients recruited from community pharmacies in Iași, Romania. Data were collected using a structured 27-item questionnaire addressing demographics, disease management, adherence, lifestyle factors, and complications. **Results:** Most participants had type 2 diabetes. Engagement in healthy behaviors was suboptimal, with low levels of daily physical activity and limited routine glucose monitoring. Complications and treatment-related side effects were frequent, reflecting a high disease burden. Treatment adherence was significantly higher among younger patients, those with type 1 diabetes and individuals who had set long-term health goals. The use of digital technologies was low overall but more common in younger participants. **Conclusions:** This study identifies critical gaps in lifestyle practices, adherence and technology uptake among Romanian patients with diabetes. These findings carry important clinical implications, as poor adherence and limited self-management are closely linked to complications and higher healthcare spending. The findings suggest that patient education, integrating validated digital tools and enhancing the role of community healthcare providers could support better adherence and reduce long-term complications.

## 1. Introduction

In addition to being considered mainly a metabolic disease, diabetes mellitus is becoming more widely acknowledged as one of the most pressing global health issues [1]. This condition is widely known for its impact on blood sugar regulation, with prolonged hyperglycemia progressively damaging blood vessels and nerves, thus increasing the risk of severe complications such as cardiovascular diseases, kidney failure, and neuropathy, and ultimately lowering the quality of life [2,3].

Globally, diabetes is among the fastest-growing health concern of the 21st century [4]. According to the 2021 IDF Diabetes Atlas estimated that 10.5% of adults aged 20 to 79 are living with diabetes, with nearly half being unaware of their condition [5]. By 2045, projections suggest that approximately 1 in 8 adults—around 783 million people—will be affected by diabetes, marking a 46% increase. Over 90% of cases are represented by type 2 diabetes, influenced by socioeconomic, demographic, environmental and genetic factors [6].

In Romania, these global trends are mirrored in worrying patterns. According to the 2023 Diabetes Data Barometer, 1 in 12 adults is living with diabetes, with only 1% of the national healthcare budget being allocated to prevention, which is among the lowest levels in the European Union (EU) [7]. Evidence from international settings shows that structured preventive programs and coordinated primary care reduce potentially avoidable hospitalizations and improve safety [8]. Despite its serious long-term complications, diabetes is often perceived as less serious than other conditions such as cancer, stroke, obesity, heart disease, arthritis or hepatitis. Alarmingly, between 20% and 30% of Romanians are at high or very high risk of developing diabetes in the next 10 years, with a higher prevalence in urban areas [9].

Currently, 1.5 million patients are registered in the National Diabetes Program, managed by the National Health Insurance House. However, the absence of a comprehensive national diabetes registry limits Romania’s ability to monitor cases efficiently and implement large scale public health measures [7]. Presently, diabetes data is tracked through a subnational registry, but efforts are underway to establish a national registry. This initiative aims to consolidate patient records, enhance disease monitoring and optimize diabetes management, ultimately providing stronger support for both patients and healthcare professionals [10].

International experience demonstrates the value of such registries. Countries such as Sweden, Denmark, Norway, the Netherlands, and the United States have well-established national databases that provide valuable insights into diagnosis, complications and treatment outcomes. For example, Sweden’s National Diabetes Register covers 90% of patients and integrates inpatient and outpatient records, while also including patient-reported metrics since 2017 [11]. Similarly, Scotland’s diabetes database systematically collects primary care data, allowing real-time adjustments in patient management [12]. These registries improve patient care, inform clinical strategies and support evidence-based policymaking [11,12]. Implementing a similar framework in Romania could significantly enhance disease monitoring, support more informed clinical decisions and ensure a more efficient use of healthcare resources.

At European level, underdiagnosis remains a critical challenge. Around 21.9 million have yet to receive a diagnosis, which represents 35.7% of the diabetic population, emphasizing the need for improved screening and awareness efforts [13]. In Romania, approximately half of individuals with diabetes remain undiagnosed, further underscoring the urgency of strengthening diagnostic strategies [7].

Such data draws attention to the critical need for more effective and early diagnostic approaches in order to reduce the risk of diabetes-related complications in Romania. Healthcare professionals play a significant role in supporting their patients in adopting healthier lifestyles, maintaining balanced diets and adhering to prescribed treatments, all of which are essential for optimal disease management and overall quality of life. Understanding patients’ knowledge, behaviors and adherence patterns is highly relevant in clinical practice, as it improves therapeutic strategies, helps prevent costly hospitalizations and supports better long-term outcomes. These real-world insights are essential for tailoring patient-centered interventions and for guiding healthcare professionals in daily clinical decision-making, consistent with evidence that high-performing healthcare systems integrate evidence-based practice with patient-centered approaches to optimize outcomes [14].

The aim of this non-interventional observational study is to assess patients’ knowledge of diabetes, their adherence to treatment and the role that lifestyle factors play in disease management. Patients with diabetes participated by completing a structured 27-item questionnaire that was distributed in community pharmacies in Iași. This survey not only served as an efficient method for data collection but also provided valuable insights into patients’ understanding of diabetes, treatment adherence, lifestyle habits and dietary practices. By identifying deficiencies in patient education, the study highlights the need for targeted interventions to enhance the quality of life for individuals living with diabetes. Our findings align with existing evidence on the importance of structured education in enhancing treatment adherence and disease management.

Unlike previous studies, which primarily focused on clinical settings, this research was conducted in community pharmacies, offering a more real-world perspective on how patients manage diabetes in their daily lives. By assessing both knowledge gaps and treatment adherence, this study provides deeper insights into the challenges patients face in managing their condition. From a healthcare delivery perspective, identifying patients with poor adherence or unhealthy lifestyle patterns early, such as in community pharmacies, can help prevent progression to severe complications requiring hospitalization. This strengthens the clinical value of such observational data [15].

Additionally, this study explores patients’ preferences regarding educational resources and digital tools, underlining the increased role of technology in diabetes self-management. These results not only highlight existing barriers but also offer valuable guidance for developing patient-centered interventions that align with real-world needs and behaviors. Importantly, these insights carry clear clinical implications: stronger patient education and better adherence are consistently linked with improved glycemic control, fewer hospitalizations and a lower risk of long-term complications such as cardiovascular disease, kidney failure, or retinopathy. Bringing real-world evidence from community settings into clinical practice can therefore reduce healthcare costs and ultimately enhance the quality of life of patients with diabetes [16].

## 2. Materials and Methods

### 2.1. Study Design

This research was conducted as a non-interventional observational study using a structured questionnaire addressed to people living with diabetes. The study aimed to assess patients’ knowledge, adherence to treatment and lifestyle habits related to disease management. Data were collected in community pharmacies in Iași, Romania, between 1 April and 1 October 2024. A custom questionnaire was developed by the authors for the purposes of this study, rather than adapted from an existing validated tool. The complete version of the patient questionnaire is available in the Supplementary Materials. Items were formulated based on the relevant literature and the research team’s clinical experience in diabetes management, with the aim of covering key aspects related to lifestyle, treatment adherence and patient perceptions. Although no formal pilot testing was conducted, the questionnaire was internally reviewed to ensure clarity and coherence. 

### 2.2. Objectives

The primary objective was to evaluate patients’ awareness of diabetes management and the importance of lifestyle modifications. The secondary objective was to analyze treatment adherence patterns and identify diabetes-related complications.

### 2.3. Study Population

The study included 100 patients who met the following eligibility criteria: ≥18 years, a confirming diagnosis of diabetes mellitus, and provision of written informed consent. Exclusion criteria included psychiatric conditions affecting judgment, refusal to participate, incomplete questionnaires, or failure to meet the inclusion criteria.

### 2.4. Data Collection Tool

The structured questionnaire comprised 27 items covering the following domains: demographics and diabetes type (age, sex, occupational status and type of diabetes), family history and comorbidities, monitoring practices (frequency of blood glucose monitoring and familiarity with long-term management strategies), lifestyle and behavioral factors (self-reported dietary habits, physical activity levels, alcohol and tobacco use, and understanding of body mass index, BMI), medication adherence and additional challenges (type of medication used, side effects experienced and difficulties in adhering to treatment regimens), patient education and psychological impact (awareness of hypoglycemia and hyperglycemia, use of modern technologies and the emotional impact of diabetes, including stressors related to the COVID-19 pandemic), and patient preferences (interest in additional resources such as dietary advice, exercise recommendations, psychological support and digital tools for better diabetes management).

The questionnaire required approximately 10 min to complete in the pharmacy setting. To encourage patient reflection, it also provided brief information on BMI calculation and its relevance for diabetes control.

### 2.5. Statistical Analysis

Data analysis was conducted using IBM SPSS Statistics version 26 (IBM Corp., Armonk, NY, USA). Descriptive statistics summarized demographic data while inferential tests were applied to explore relationships between variables. Non-parametric tests, including Mann–Whitney U and Kruskal–Wallis, were applied for ordinal data, while Spearman’s correlation coefficient assessed associations between variables. Categorical variables were analyzed using chi-square tests. A significance level of *p* < 0.05 was set for statistically significant.

### 2.6. Ethical Considerations

The study was conducted in accordance with the Declaration of Helsinki and approved by Ethics Commission of University of Medicine and Pharmacy ‘Grigore T. Popa’ Iași (No. 405/28.02.2024). Participants received detailed information about the study’s purpose, procedures and their rights. Participation was voluntary and patients could withdraw consent at any time before October 1st, 2024, without any impact on their medical care. No financial incentives were provided. Confidentiality was strictly maintained, with all personal data anonymized and securely stored.

## 3. Results

### 3.1. Demographic and Clinical Characteristic

The study sample consisted of 100 participants, of whom 35% identified as male, 41% as female, and 24% did not disclose their gender. This unusually high proportion of missing data is acknowledged as a limitation and may reflect either participant oversight or reluctance to disclose this information. The largest age group was 50–59 years old (27%), followed by 40–49 years (20%) and 60–69 years (18%), with only 3% being under 30 years. Regarding occupational status, 49% of participants were retired, while 47% were employed, 1% student and 3% homemakers.

Most participants (69%) had type 2 diabetes, 28% had type 1 diabetes, and 3% reported gestational diabetes. A family history of diabetes was noted in 61% of participants, while 36% indicated no family history and 3% did not respond. The detailed demographic and clinical characteristics of the sample population are provided in Table 1a,b.

Among patients with type 1 diabetes, 60.7% reported a family history of the disease, compared with 59.4% of those with type 2 diabetes. All three participants with gestational diabetes reported a positive family history (Table 2).

### 3.2. Self-Care Behaviors

Daily blood glucose monitoring was reported by 31% of participants, while 30% monitored multiple times per day, 27% several times per week and 12% only once a week.

In terms of diet, 59% of participants considered their diet “healthy” and 20% “very healthy,” while 17% described it as “unhealthy.” Physical activity was limited: 21% exercised daily, 19% several times per week, 38% occasionally and 22% not at all.

Most participants abstained from alcohol (51%), while 48% consumed it occasionally and 1% frequently. Coffee consumption was common (59%), while 41% did not consume coffee. Table 3 presents a detailed summary regarding participants’ diabetes management.

### 3.3. Treatment Patternes and Complications

Table 4a–c presents an overview of the participants’ treatment options, including treatment duration, effectiveness, tolerability and the frequency of complications or medication adjustments due to side effects. The majority of participants (59%) were prescribed oral antidiabetic medication, while 19% relied on insulin therapy and 18% used a combination of both. A significant proportion (57.9%) had been on treatment for more than two years, while only 6.3% had started therapy within the past six months (Table 4a).

Patient perceptions of medication effectiveness were generally positive: more than three quarters rated their therapy as “good” or “very good.” However, 45.8% of patients reported experiencing side effects, and in 43.8% of cases these adverse effects led to treatment modifications (Table 4b).

In terms of diabetes-associated complications, two-thirds of participants (66.3%) reported at least one complication, while only one-third indicated no complications (Table 4c).

**Figure 1:**
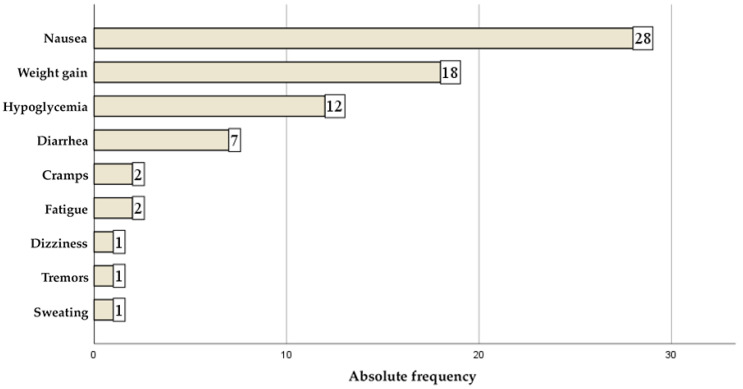
Side effects associated with diabetes treatment, reported by respondents.

Patient perception of medication was generally positive: 55.8% rated treatment as “good” and 23.2% as “very good.” Side effects were reported by 45.8% of patients, most frequently nausea (28 cases), weight gain (18 cases) and hypoglycemia (12 cases). Less frequent events included diarrhea (7 cases), cramps and fatigue (2 cases each) and dizziness, tremors, and sweating (1 case each) (Figure 1).

**Figure 2:**
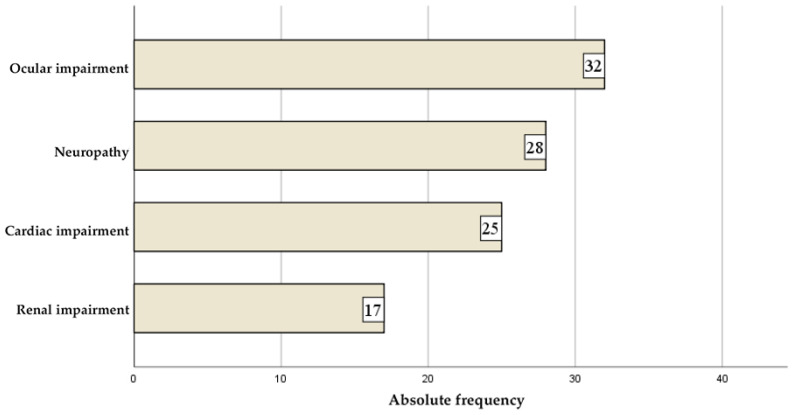
Diabetes-related complications.

Complications were common, with ocular manifestations (32 cases), neuropathy (28 cases), and cardiac complications (25 cases) being the most frequently reported (Figure 2). Detailed treatment patterns are presented in Table 4.

More than half of participants (57%) were aware of their abdominal circumference, while 43% were not. Only 43.2% had established long-term goals for diabetes management. Most participants (75%) did not use digital technologies in disease management, as summarized in Table 5.

### 3.4. Treatment Adherence

Adherence to treatment was assessed using the question, “How easy is it for you to follow your medication schedule?”. Participants’ responses were analyzed as mean ± standard deviation, given their ordinal nature and similarity to a Likert scale. Since the variable did not follow a normal distribution, the Mann–Whitney U test was applied for comparisons between two groups and the Kruskal–Wallis test was used for comparisons across three or more groups.

Participants under 40 years of age demonstrated the highest adherence (mean = 3.80, SD = 0.42), compared to those aged 40–60 years (mean = 2.84, SD = 0.88) and those over 60 years (mean = 2.71, SD = 0.87), with a statistically significant difference (*p* = 0.002). This may reflect greater adaptability and medication management among younger individuals compared to older age groups, where adherence tends to decline.

When analyzed by diabetes type, participants with type 1 diabetes demonstrated higher adherence (mean = 3.18, SD = 0.94) compared to those with type 2 diabetes (mean = 2.76, SD = 0.85), with a significant difference (*p* = 0.025). This result could be attributed to the nature of type 1 diabetes, which requires constant monitoring and strict glycemic control, in contrast to type 2 diabetes, where treatment regimens may be more flexible.

Adherence was significantly higher among individuals who had set long-term goals for diabetes management (mean = 3.33, SD = 0.70) compared to those without such goals (mean = 2.53, SD = 0.89), with a highly significant difference (*p* < 0.001). These findings suggest that planning and setting long-term goals encourages patients to maintain higher levels of motivation and responsibility for their health.

Although no significant differences in adherence were identified between genders (*p* = 0.206), women reported slightly higher adherence on average (mean = 3.05) compared to men (mean = 2.74).

In addition to *p*-values, effect sizes were calculated (η^2^) to provide further context regarding the strength of these associations. The largest effect was observed for long-term goal setting (η^2^ = 0.20), indicating a substantial influence on adherence, while the effect sizes for age (η^2^ = 0.13) and diabetes type (η^2^ = 0.05) were moderate, as summarized in Table 6.

### 3.5. Use of Digital Technologies

Adoption of digital technologies differed significantly across age groups (*p* < 0.001). Among participants < 40 years, 83.3% used modern tools for diabetes management, compared with 29.8% of those aged 40–60 and only 2.4% of those over 60 years. Use was also significantly higher among type 1 diabetes patients (50%) compared with type 2 (13%) (*p* < 0.001), as presented in Table 7. 

### 3.6. Long-Term Goal Setting

Patients with type 1 diabetes were more likely to establish long-term goals (56.0%) compared with type 2 (36.8%), although the difference was not statistically significant (*p* = 0.096). The presence of comorbidities significantly reduced the likelihood of goal setting (*p* = 0.014), while the presence of complications showed only a non-significant trend (*p* = 0.091), as presented in Table 8.

### 3.7. Psychological and Emotional Impact

The psychological and emotional burden of diabetes was assessed based on diabetes type, with differences between groups evaluated using the chi-square test (*p* = 0.045), indicating a statistically significant difference.

Patients with type 1 diabetes reported a lower overall impact, with 35.7% indicating no effect, while only 3.6% experienced a severe impact. The majority (60.7%) reported a mild impact. In contrast, patients with type 2 diabetes faced a greater emotional burden, with 24.6% experiencing a severe impact, 52.2% reporting a mild impact, and 23.2% stating they were not emotionally affected, as summarized in Table 9.

Correlation analysis showed that higher physical activity (r = –0.270, *p* = 0.007), frequent reading of food labels (r = –0.199, *p* = 0.050), and better treatment adherence (r = –0.334, *p* = 0.001) were significantly associated with lower psychological burden. No significant association was found between blood glucose monitoring and psychological impact (*p* = 0.256), as illustrated in Table 10.

## 4. Discussion

This observational study conducted in community pharmacies in Iași provides real-world insight into lifestyle behaviors, treatment adherence, psychological burden and the uptake of digital tools among Romanian patients with diabetes. According to the National Report on the Health Status of the Romanian Population 2020, chronic diseases are systematically recorded in family medicine practices, highlighting three major conditions: hypertensive heart disease, ischemic heart disease and diabetes mellitus [17]. Romania continues to report very low rates of regular exercise and fruit or vegetables consumption compared with the EU average, which is consistent with the lifestyle gaps documented in our cohort [9].

Lifestyle behaviors were observed to be strongly associated with indicators of disease control. Only 21% of participants reported daily physical activity; whereas 22% reported none. Combined with suboptimal dietary practices, these findings are compatible with the high prevalence of obesity and cardiometabolic complications we recorded (cardiovascular disease, neuropathy, retinopathy and nephropathy) and emerging comorbidities such as metabolic dysfunction-associated steatotic liver disease and cognitive decline [16]. These findings align with European outpatient cohorts of elderly patients, where despite reported adherence, overall cardiometabolic control remains unsatisfactory: only a minority met targets for HbA1c, fasting glucose, or blood pressure goals [18].

In addition to suboptimal metabolic control, nearly half (45.8%) of patients reported treatment-related side effects, most commonly nausea, weight gain and hypoglycemia. Such adverse effects, when combined with poor adherence, have clear clinical relevance, as they increase the risk of complications and place a heavier demand on healthcare services. Prior evidence indicates that nonadherence to oral agents was associated with nearly twice the odds of all-cause hospitalization and increased costs, underscoring the clinical and economic implications of poor therapeutic engagement [19,20].

In our study, adherence did not differ significantly by gender (*p* = 0.206), although women reported slightly higher mean adherence than men. This finding is in line with broader trends in the EU, where women were more likely than men to use prescribed medicines in 2019, according to the latest Eurostat data [21]. At the same time, other studies suggest a more complex reality, where the female gender has been identified as an independent predictor of lower adherence to antidiabetic treatment, with women experiencing worse clinical outcomes. These gender differences in medication adherence are influenced by multiple factors, including sociodemographic aspects, disease burden, treatment complexity, and psychological influence [22]. However, existing evidence on gender-related differences in adherence remains limited, largely due to the absence of large-scale, comprehensive studies.

Our results indicate a strong association between healthy behaviors and improved diabetes management. Participants who maintained a healthy or very healthy diet were more likely to adhere to their treatment plans and reported lower emotional distress related to diabetes. Conversely, those with unhealthy eating habits and minimal physical activity were more frequently associated with difficulties in managing their condition and higher psychological stress. Healthcare professionals must focus on prioritizing initiatives that motivate patients to adopt and sustain healthy behaviors.

Our study also explored the psychological and emotional impact of diabetes. A statistically significant difference (*p* = 0.045) was noted, revealing that most individuals with type 1 and type 2 diabetes experience some level of emotional distress. However, the intensity of this burden was notably lower among those with type 1 diabetes. The majority (35.7%) reported minimal distress, whereas only 3.6% described their experience as extremely challenging. In contrast, nearly a quarter (24.6%) of type 2 diabetes patients indicated extreme psychological distress. One possible explanation for this disparity is that individuals with type 1 diabetes adapt to their condition from an early age, fostering stronger coping mechanisms. Meanwhile, type 2 diabetes is often diagnosed later in life, requiring major lifestyle changes that may contribute to heightened emotional distress.

A Romanian study assessed the prevalence of depression among type 2 diabetes patients using the Patient Health Questionnaire-9 (PHQ-9) yielded similar findings. While 51.4% of patients showed no signs of depression, the remaining participants exhibited varying levels of depression symptoms. Specifically, 19.6% reported mild depression, 17.8% moderate, 6.5% moderate-severe, and 4.7% severe. The high prevalence of depression symptoms was associated with metabolic imbalances requiring hospitalization, as well as with difficulties in managing chronic complications [23]. Taken together, these results reinforce that psychological well-being is integral to diabetes care and closely linked to adherence and self-care capacity [24,25,26].

In our study, the results showed that long-term goal setting was strongly associated with higher treatment adherence. Participants who had long-term treatment goals exhibited significantly higher treatment adherence (mean = 3.33, SD = 0.70) compared to those without goals (mean = 2.53, SD = 0.89), with a very strong statistical significance (*p* < 0.001). These results suggest a potential role for structured goal-setting in patient motivation and active involvement in their care. This aligns with guidance that positions goal setting as a key strategy in diabetes self-management strategy and with evidence that structured goal-setting interventions can reduce HbA1c levels and improve glycemic control [27,28]. In the Romanian context, these results support the systematic incorporation of goal-oriented counseling into routine encounters in primary care and community pharmacy [15,29].

Technology adoption in our study was low overall (25%) and was strongly age-dependent especially among younger adults and people with type 1. Evidence from previous studies suggests that well-validated digital solutions can complement self-management and adherence, but their successful use in Romania will depend on ensuring equity and tailoring interventions to the needs of seniors and lower-income groups [30,31].

A recent survey among Romanian patients with type 1 diabetes explored the use of CGM systems, insulin pumps and other diabetes management technologies. The findings revealed that these devices are now a key part of daily care, helping many patients feel more confident in their treatment decisions. Some people have adopted them easily, making them a part of their daily routine. However, concerns remain regarding device accuracy, sensor lifespan and accessibility through the national healthcare system [32]. Aligning national practice with international guidance may strengthen clinical outcomes: the ADA Standards of Care 2025 emphasize individualized HbA1c targets (<7% for many adults) and CGM-based metrics (such as higher Time-in-Range with minimized hypoglycemia) as actionable clinical endpoints [33,34].

The present study has several limitations that should be considered when interpreting the findings. The analysis was based on data from 100 participants recruited in Iași, a single urban center, which may limit the extent to which these results can be generalized to the broader Romanian population. The predominantly urban background of respondents may have influenced their adherence patterns, access to medical information, and use of digital tools for diabetes management. Future investigations should aim to include larger and more geographically diverse samples to improve external validity and ensure representativeness across different settings. Moreover, the questionnaire used in this research was newly designed by the authors and has not yet undergone validation or pilot testing, which could affect the reproducibility of certain results. As with all self-reported surveys, recall and social desirability biases cannot be fully excluded. From a statistical perspective, non-parametric tests were applied after confirming the non-normal distribution of variables; however, no adjustments for multiple testing were made. As the analyses were hypothesis-oriented and exploratory in nature, the findings should be interpreted cautiously with respect to potential type I error. These factors may have introduced bias into the results and future studies with larger, more diverse samples and objective measures would help strengthen the evidence.

Despite these constraints, this study adds context-specific evidence from Romania and underscores three actionable levers: (i) closing lifestyle gaps related to diet and physical activity; (ii) addressing psychological burden as a routine element of care; (iii) embedding structured goal setting into patient education. These patient-level levers, coupled with frontline delivery in community pharmacy and primary care, can serve as pragmatic foundations for improving adherence and reducing complications [7,15,18,35,36]. System-level considerations (such as digital health integration, national registries and reimbursement policies) are considered separately in the Implications for Practice section.

### Implications for Practice

These findings of this study highlight several priorities for strengthening diabetes care in Romania. While the results confirm global trends regarding lifestyle, adherence and psychological burden, they also identify context-specific opportunities for intervention at patient, provider and system level.

1. The Role of Community Pharmacies in Screening and Education

A nationwide pharmacy-based pilot project have already demonstrated that community pharmacies can play a decisive role in diabetes prevention [35]. In one such program, more than 20% of participants face a high or very high risk of developing diabetes within the next ten years [7]. Through the use of a standardized risk assessment questionnaire, pharmacists identified individuals at-risk and provided targeted educational guidance on preventive strategies [35]. The final analysis of over 10,000 individuals, indicated that 22% were high-risk, emphasizing the urgent need for early screening programs [7,35]. Moreover, pharmacist-led interventions such as individualized 25–30 min counseling sessions on insulin administration and self-monitoring, provision of educational booklets covering diet, exercise and hypoglycemia management, and follow-up phone support have been shown to improve glycemic control and adherence [36,37]. These experiences suggest that community pharmacies could be systematically integrated into national strategies for early detection, education and adherence monitoring.

2. Patient Education and Goal Setting

The strong association in our study between long-term goal setting and treatment adherence (*p* < 0.001) underscores the practical value of structured patients’ education. Beyond generic advice, goal-oriented counseling provides concrete behavioral targets that motivate patients and sustain engagement. Integrating such interventions into routine care, whether in primary care or pharmacy settings, could be a cost-effective strategy to improve outcomes in Romania. Consistent with international evidence, embedding goal setting in counseling protocols should be considered a priority in national diabetes programs [38,39].

3. Addressing Psychological Burden

Nearly one quarter of patients with type 2 diabetes in our study reported extreme psychological distress, a finding consistent with Romanian data using PHQ-9 [23]. This highlights the need to systematically incorporate psychological screening and support into routine diabetes care. Practical steps could include regular assessment of distress, early referral to psychological services and training for healthcare providers to identify emotional challenges. Addressing psychological burden is not only essential for patient well-being but also directly influences adherence and disease control [28,29,40,41,42].

4. Digital Health and Technology Integration

Digital health solutions remain underutilized in Romania, with only 25% of our participants reporting use, predominantly younger patients and those with type 1 diabetes. This age-dependent digital divide highlights inequities in access and digital literacy. Validated tools such as continuous glucose monitoring (CGM), insulin pumps, and mobile health applications can improve glycemic control, treatment adherence, and patient confidence. AI-powered solutions, such as Diabeta, Pharmachat and Chat4Doc, are already available and could support both clinicians and patients in treatment decision-making. To ensure equitable access, policies should focus on reimbursement, patient training, and digital literacy programs tailored especially to older adults [30,32,43,44,45,46].

5. System-Level Reforms and Policy Priorities

Romania continues to face high rates of diabetes-related complications and hospitalizations, reflecting persistent gaps in prevention, adherence, and access to modern care. To address these challenges, several system-level reforms are needed: expansion of reimbursement policies for advanced technologies (CGM, insulin pumps) to reduce inequities in access [47]; implementation of the National Diabetes and Prediabetes Registry, which would improve disease tracking and facilitate data-driven policy [48]; reinstatement of structured national screening and prevention programs, such as the discontinued National Programme for the Evaluation of Population’s Health [49]; integration of digital health into standard care pathways, modeled after successful examples in Sweden and the Netherlands [50,51]. By aligning with best practices from other EU countries, Romania can modernize diabetes care, reduce avoidable hospitalizations, and lower long-term healthcare costs.

Experiences from neighboring countries further highlight the value of structured prevention and system-level reforms. In Bulgaria, the National Programme for the Prevention of Chronic Non-Communicable Diseases 2021–2025 has set measurable targets to reduce smoking, increase physical activity, and achieve declines in type 2 diabetes and hypertension prevalence, demonstrating how clear population-level objectives can shape national health policy [52]. In Hungary, the Ormánság Health Program illustrates the urgent need for targeted interventions in socially deprived regions, where both diagnosed and undiagnosed diabetes rates exceed the national average, and where universal health insurance has played a key role in facilitating access to therapy despite socioeconomic barriers [53]. Serbia offers another instructive model: the development of a Book of Electronic Diabetes Records, coordinated nationally and linked with strengthened primary-care diabetes units, has improved routine monitoring (HbA1c checks, microvascular screening), streamlined referrals, and supported the integration of lifestyle interventions into everyday practice [54].

By aligning with best practices from other EU countries, Romania can modernize diabetes care, reduce avoidable hospitalizations and lower long-term healthcare costs. A recent Romanian study highlighted the substantial financial impact of diabetes complications, which often result in longer hospital stays and increased treatment expenses [55]. Despite the emphasis placed on structured patient education in the 2021 Diabetes Management Guidelines, access to standardized programs remains limited [56]. Although a subnational diabetes registry currently exists, efforts to establish a nationwide Diabetes and Prediabetes Registry are ongoing and would represent a critical step forward [48].

6. Recommendations for Romania

Based on the results of this study and existing evidence, several concrete priorities emerge for Romanian diabetes care: expand community pharmacy involvement in nationwide screening and education programs; incorporate structured goal-setting interventions into routine counseling; introduce systematic psychological screening and support for patients with type 2 diabetes; improve reimbursement and digital literacy to ensure equitable adoption of validated technologies and implement the National Diabetes and Prediabetes Registry to strengthen disease monitoring and healthcare planning.

## 5. Conclusions

The present study underlines the urgent need for precise actions to strengthen diabetes care in Romania. Expanding funding for prevention and education may represent a critical step to support awareness campaigns and structured lifestyle programs. Establishing a national diabetes registry would provide a necessary framework for systematic disease monitoring and early intervention. Equally, integrating digital health tools such as CGM-based metrics and mobile applications could help bridge gaps in access and promote self-management, particularly in underserved populations.

In summary, strengthening patient support through structured education, adherence monitoring and psychological counseling in both primary care and pharmacy practice is essential to improve outcomes. By implementing these priorities, Romania can modernize its diabetes care system, reduce preventable complications and enhance both quality of life and cost-effectiveness.

## Figures and Tables

**Table 1:** (a) Demographic characteristics and diabetes type of the studied population (*n* = 100). (b) Clinical Characteristic Of the Study Population (*n* = 100).

(a)			
		*n*	%
Gender	Male	35	35.0%
	Female	41	41.0%
	Not specified	24	24.0%
Age (years)	<30	3	3.0%
	30–39	9	9.0%
	40–49	20	20.0%
	50–59	27	27.0%
	60–69	18	18.0%
	70–79	16	16.0%
	80+	7	7.0%
Employment status	Student	1	1.0%
	Employed	47	47.0%
	Retired	49	49.0%
	Homemaker	3	3.0%
**(b)**			
		***n***	**%**
Diabetes classification	Type 1	28	28.0%
	Type 2	69	69.0%
	Gestational	3	3.0%
Diabetes family history	No	36	36.0%
	Yes	61	61.0%
	Not specified	3	3.0%

**Table 2:** Diabetes classification.

Diabetes Classification	Diabetes Family History	*n*	%
Type 1	No	11	39.3%
	Yes	17	60.7%
Type 2	No	25	36.2%
	Yes	41	59.4%
Gestational	No	0	0.0%
	Yes	3	100.0%

**Table 3:** Self-care behavior among participants with diabetes.

		*n*	%
Blood Glucose Monitoring	Once a week	12	12.0%
	Several times a week	27	27.0%
	Once a day	31	31.0%
	Multiple times a day	30	30.0%
Diet	Unhealthy	17	17.0%
	Healthy	59	59.0%
	Very healthy	20	20.0%
Physical Activity	None	22	22.0%
	Occasionally	38	38.0%
	Several times per week	19	19.0%
	Daily	21	21.0%
Alcohol	None	51	51.0%
	Occasionally	48	48.0%
	Frequently	1	1.0%
Coffee consumption	No	41	41.0%
	Yes	59	59.0%
Smoking	No	79	79.0%
	Yes	21	21.0%

**Table 4:** (a) Treatment patterns. (b) Patient perceptions and side effects. (c) Diabetes-associated complications.

(a)			
		*n*	%
Treatment type	None	4	none
	Oral antidiabetics	59	59.0%
	Insulin	19	19.0%
	Both oral antidiabetics and insulin	18	18.0%
Duration of treatment	>6 months	6	6.3%
	6 months to 1 year	14	14.7%
	1–2 years	20	21.1%
	More than 2 years	55	57.9%
**(b)**			
		***n***	**%**
Patient perception of medication	Not good	1	1.0%
	Neutral	19	20.0%
	Good	53	55.8%
	Very good	22	23.2%
Reported side effects	No	52	54.2%
	Yes	44	45.8%
Changes in medication due to side effects	No	54	56.2%
	Yes	42	43.8%
**(c)**			
Reported diabetes complications	No	33	33.7%
	Yes	65	66.3%

**Table 5:** Patient Self-Management Awareness and Digital Tool Adoption.

Question	Answer	*n*	%
Are you aware of your abdominal circumference?	No	43	43.0%
	Yes	57	57.0%
Have you set long-term goals for managing your diabetes?	No	54	56.8%
	Yes	41	43.2%
Do you use modern technologies in diabetes management?	No	75	75.0%
	Yes	25	25.0%

**Table 6:** Treatment Adherence According to Demographic and Clinical Characteristics.

		Treatment Adherence		*p*	
		Mean	SD		Effect Size (η^2^)
Gender	Male	2.74	(1.01)	0.206	0.02
	Female	3.05	(0.84)		
Age	<40	3.80	(0.42)	0.002 *	0.13
	40–60	2.84	(0.88)		
	60+	2.71	(0.87)		
Diabetes classification	Type 1	3.18	(0.94)	0.025 *	0.05
	Type 2	2.76	(0.85)		
Long-term goals for diabetes management	No	2.53	(0.89)	0.000 *	0.20
	Yes	3.33	(0.70)		

* Statistically significant correlations (*p* < 0.05).

**Table 7:** Use of Modern Technologies for Diabetes Management by Age Group and Diabetes Type.

		Do You Use Modern Technologies for Diabetes Management?				*p*	
		NO		YES			
		*n*	%	*n*	%		Effect Size (Cramér’s V)
Age	<40	2	16.7%	10	83.3%	0.000 *	0.58
	40–60	33	70.2%	14	29.8%		
	60+	40	97.6%	1	2.4%		
Diabetes classification	Type 1	14	50.0%	14	50.0%	0.000 *	0.40
	Type 2	60	87.0%	9	13.0%		

* Statistically significant correlations (*p* < 0.05).

**Table 8:** Association of Long-Term Goal Setting for Diabetes Management with Clinical Characteristics.

		Did You Set Long-Term Goals for Diabetes Management?				*p*	
		NO		YES			
		*n*	%	*n*	%		Effect Size (Cramér’s V)
Diabetes classification	Type 1	11	44.0%	14	56.0%	0.096	0.17
	Type 2	43	63.2%	25	36.8%		
Comorbidities	No	11	37.9%	18	62.1%	0.014 *	0.25
	Yes	43	65.2%	23	34.8%		
Complications	No	14	45.2%	17	54.8%	0.091	0.17
	Yes	40	63.5%	23	36.5%		

* Statistically significant correlations (*p* < 0.05).

**Table 9:** Impact of Diabetes on Psychological and Emotional Status by Diabetes Type.

		How Has Diabetes Affected Your Psychological and Emotional State?						*p*	Effect Size (Cramér’s V)
		Not Affected		Mildly Affected		Severely Affected			
		*n*	%	*n*	%	*n*	%		
Diabetes type	Type 1	10	35.7%	17	60.7%	1	3.6%	0.045 *	0.25
	Type 2	16	23.2%	36	52.2%	17	24.6%		

* Statistically significant correlations (*p* < 0.05).

**Table 10:** Correlation Between Psychological/Emotional Impact and Self-Management Behaviors in Diabetes.

How Has Diabetes Affected Your Psychological and Emotional State?			
Correlation coefficient		*p*	
Blood glucose monitoring		0.256	−0.115
Physical activity		0.007 *	−0.270
Frequency of reading food labels		0.050 *	−0.199
Treatment adherence		0.001 *	−0.334

* Statistically significant correlations (*p* < 0.05).

## Data Availability

The original contributions presented in this study are included in the article.

## Supplementary Materials

The following supporting information can be downloaded at: https://www.mdpi.com/article/10.3390/jcm14207171/s1. Supplementary File S1: Full version of the patient questionnaire used in the study.
