# Supplementary material for: Real-World and Clinical Implications of Patient Education, Lifestyle and Treatment Adherence in Romanian Diabetes Care: An Observational Study
Source: J Clin Med. 2025 Oct 11;14(20):7171. doi: 10.3390/jcm14207171 (PMC12564956; doi:10.3390/jcm14207171)
Supplement: Supplementary file 1 [file jcm-14-07171-s001.zip › jcm-3904353-supplementary.pdf]

Chestionar

Vârsta: ________ Sex: M/F **Status ocupațional**: ☐ student ☐ salariat ☐ pensionar

1. Tipul de diabet:

- ☐ Tip 1
- ☐ Tip 2
- ☐ Gestațional
- ☐ Altul

2. Există cazuri de diabet în familie?

- ☐ Da
- ☐ Nu

3. Cât de des vă măsurați glicemia?

- ☐ De mai multe ori pe zi
- ☐ O dată pe zi
- ☐ De câteva ori pe săptămână
- ☐ O dată pe săptămână

4. Cum ați clasifica dieta dvs. actuală?

- ☐ Foarte sănătoasă
- ☐ Sănătoasă
- ☐ Nesănătoasă

5. Cât de des faceți exerciții fizice?

- ☐ Zilnic
- ☐ De câteva ori pe săptămână
- ☐ Ocazional
- ☐ Niciodată

6. Cât de des consumați alcool?

- ☐ Des
- ☐ Ocazional
- ☐ Deloc

7. Consumați regulat cafea sau tutun?

- ☐ Cafea
- ☐ Tutun
- ☐ Ambele
- ☐ Niciunul

8. Suferiți de alte boli asociate?

- ☐ Hipertensiune arterială
- ☐ Boli cardiovasculare
- ☐ Nu

Altele: ……………………………………………………….

9. Ce tip de medicație utilizați pentru diabet?

- ☐ Insulină
- ☐ Medicamente orale
- ☐ Ambele
- ☐ Niciuna

10. De cât timp urmați actualul tratament medicamentos pentru diabet?

- ☐ > 6 luni
- ☐ 6 luni - 1 an
- ☐ 1-2 ani
- ☐ > 2 ani

11. Cum vă simțiți cu medicația pe care o luați pentru diabet?

- ☐ Foarte bine
- ☐ Bine
- ☐ Așa și așa
- ☐ Nu prea bine

12. Ați schimbat vreodată medicația pentru diabet din cauza efectelor secundare?

- ☐ Da
- ☐ Nu

13. Dacă da, care au fost acestea?

- ☐ Grețuri
- ☐ Scăderea zahărului din sânge
- ☐ Creșterea în greutate

Altele: ……………………………………….

14. Ați experimentat complicații legate de diabet?

- ☐ Da
- ☐ Nu

15. Dacă da, ce tip de complicații?

- ☐ Probleme la nivelul ochilor
- ☐ Probleme renale
- ☐ Neuropatie
- ☐ Probleme cardiace

Altele: ………………………………………………….

16. Cât de ușor este pentru dumneavoastră să respectați programul de medicație?

- ☐ Foarte ușor
- ☐ Ușor
- ☐ Nici ușor, nici dificil
- ☐ Dificil

17. Cum ați descrie comunicarea cu medicul dvs. despre opțiunile de tratament?

- ☐ Satisfăcătoare
- ☐ Acceptabilă
- ☐ Nesatisfăcătoare

18. Vă știți circumferința abdominală?

- ☐ Da
- ☐ Nu

19. Cunoașteți diferența dintre *hipoglicemie* (nivel scăzut de zahăr în sânge) și *hiperglicemie* (nivel ridicat de zahăr în sânge)?

- ☐ Da, foarte clar
- ☐ Da, în mare parte
- ☐ Nu înțeleg diferențele

20. Cât de des citiți etichetele alimentelor pentru a verifica conținutul de carbohidrați și zahăr?

- ☐ De fiecare dată
- ☐ Rar
- ☐ Niciodată

21. Ați primit informații legate de indicele de masă corporală (IMC) ?

- ☐ Da
- ☐ Nu
- ☐ Nu sunt sigur/ă
- ☐ Nu știu ce este IMC

22. Cum a afectat diabetul starea dvs. psihologică și emoțională?

- ☐ Neafectată
- ☐ Ușor afectată
- ☐ Sever afectată

23. Cum gestionați episodul de hipoglicemie (scăderea zahărului din sânge)?

- ☐ Iau rapid carbohidrați simpli (ex. zahăr, suc)
- ☐ Consum alimente cu indice glicemic ridicat
- ☐ Folosesc medicamente de urgență prescrise de către medicul meu
- ☐ Aștept să treacă de la sine

Alte metode: ....

24. Aveți stabilite obiective pe termen lung legate de managementul diabetului? (ex. menținerea anumitor niveluri ale HbA1c, prevenirea complicațiilor)

- ☐ Da
- ☐ Nu

Dacă da, care sunt:

....

25. Ce tipuri de informații sau resurse credeți că v-ar ajuta mai mult în gestionarea diabetului?

- ☐ Informații despre alimentație
- ☐ Sfaturi despre exerciții fizice
- ☐ Suport psihologic
- ☐ Tehnologii și aplicații

26. Folosiți tehnologii moderne în managementul diabetului? (ex. aplicații mobile, monitoare continue de glicemie):

- ☐ Da
- ☐ Nu

27. Cum a influențat pandemia COVID-19 gestionarea diabetului dvs.?

- ☐ Neafectată
- ☐ Ușor afectată
- ☐ Sever afectată

Mulțumim pentru completarea chestionarului!

Dorim să vă informăm că atât chestionarul, cât și calculul Indicelui de Masă Corporală (IMC) pe baza formulei menționate, sunt instrumente informative, nu metode de diagnosticare sau tratament. Pentru orice clarificări sau sfaturi legate de gestionarea diabetului sau interpretarea rezultatelor discutați direct cu medicul dvs.

Chestionar

Tu știi care este valoarea IMC-ului tău?

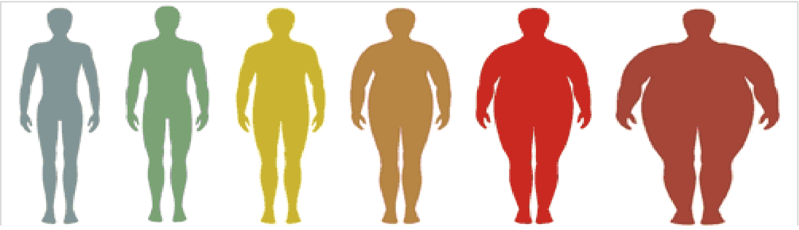

Un aspect important în gestionarea diabetului este menținerea unei greutăți sănătoase.

Vă încurajăm să vă calculați **IMC-ul** folosind următoarea formulă:

$$\mathbf{IMC} = \frac{\text{Greutatea } (\mathbf{kg})}{\text{Inălțimea}^2 (\mathbf{m})}$$

IMC	Interpretare rezultat
**18,49 sau mai puțin**	Subponderal
**18,50-24,99**	Greutate normală
**25,00-29,99**	Supraponderal
**30,00-34.99**	Obezitate (grad I)
**35,00-39,99**	Obezitate (grad II)
**40,00 sau mai mult**	Obezitate morbidă

Dorim să vă informăm că atât chestionarul, cât și calculul Indicelui de Masă Corporală (IMC) pe baza formulei menționate, sunt instrumente informative, nu metode de diagnosticare sau tratament. Pentru orice clarificări sau sfaturi legate de gestionarea diabetului sau interpretarea rezultatelor discutați direct cu medicul dvs.

Pentru a vă susține în managementul diabetului și promovarea unui stil de viață sănătos, iată câteva sfaturi generale:

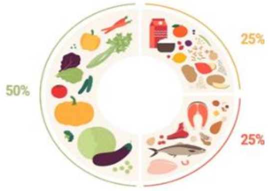

Alimentație echilibrată

Includeți în dietă o varietate de alimente bogate în nutrienți, cu accent pe legume, fructe, cereale integrale și proteine slabe.

Limitați consumul de zaharuri adăugate, grăsimi saturate și sodiu.

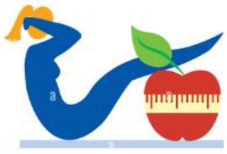

Activitate fizică

Încercați să faceți cel puțin 150 de minute de activitate fizică moderată pe săptămână, cum ar fi mersul pe jos rapid, înotul sau ciclismul.

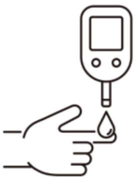

Monitorizarea glicemiei

Păstrați sub control nivelurile de zahăr din sânge prin monitorizări regulate, conform recomandărilor medicului dumneavoastră.

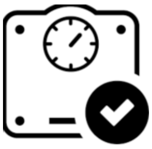

Menținerea unei greutăți sănătoase

Măsurarea **circumferinței taliei**, **determinarea greutății corporale** și a **indicelui de masa corporală** sunt foarte importante în controlul diabetului

Cum trebuie măsurată circumferința taliei?

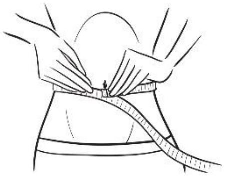

	Risc mic	Risc mare
**Bărbați**	≤ 94 cm	≥ 102 cm
**Femei**	≤ 80 cm	≥ 88 cm

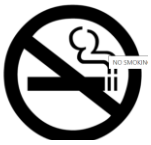

Evitarea fumatului

Fumatul poate agrava complicațiile diabetului.

Dacă fumați, căutați ajutor și consiliere pentru a renunța.

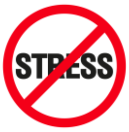

Evitarea stresului

Tehnicile de relaxare, cum ar fi meditația, yoga sau respirația profundă, pot ajuta în gestionarea stresului, care la rândul său poate influența controlul glicemiei.

Vă încurajăm să discutați aceste sfaturi și orice modificări pe care doriți să le faceți în stilul de viață cu medicul dumneavoastră, pentru a lua cele mai bune decizii adaptate nevoilor și condiției dumneavoastră de sănătate.
